# Fabrication, characterization, and kinetic study of vertical single-crystalline CuO nanowires on Si substrates

**DOI:** 10.1186/1556-276X-7-119

**Published:** 2012-02-13

**Authors:** Shao-Liang Cheng, Ming-Feng Chen

**Affiliations:** 1 Department of Chemical and Materials Engineering, National Central University, Chung-Li City, Taoyuan County, 32001, Taiwan, Republic of China

**Keywords:** Cu film, thermal oxidation, CuO nanowire, growth kinetic, resistance

## Abstract

We report here on the first study of the growth kinetics of high-yield, vertical CuO nanowires on silicon substrates produced by the process of thermal oxidation. The length of the CuO nanowires could be tuned from several to tens of micrometers by adjusting the oxidation temperature and time. The grown CuO nanowires were determined to be single-crystalline with different axial crystallographic orientations. After a series of scanning electron microscopy examinations, the average length of CuO nanowires produced at each temperature was found to follow a parabolic relationship with the oxidation time. The parabolic growth rate at different oxidation temperatures was measured. The activation energy for the growth of CuO nanowires calculated from an Arrhenius plot was found to be about 174.2 kJ/mole. In addition, the current-voltage characterization indicated that the sample with high-density CuO nanowires exhibited ohmic behavior, and its resistance was found to significantly decrease with increasing environmental temperature. The result can be attributed to an increase in the number of carriers at higher temperatures.

## Introduction

In recent years, one-dimensional [1D] metal-oxide semiconductor nanostructures, such as whiskers and nanowires, have attracted increasing interest due to their unique properties and variety of potential applications [1-3]. Among the metal oxides, cupric oxide [CuO] has been extensively studied as a p-type metal-oxide semiconductor. It has a direct bandgap of about 1.2 to 2.0 eV and exhibits many excellent physical and chemical properties [4-6]. Nanostructured CuO materials, especially 1D CuO nanowires, have received much more attention, having already been applied in field-effect transistors, photovoltaic cells, field emission nanodevices, and chemical and gas sensors [7-10]. Various growth techniques, such as the hydrothermal method [11], the thermal decomposition method [12], and the templating sol-gel method [13], have been developed to produce large-scale CuO nanowires. Recently, a simpler and more convenient route for the fabrication of CuO nanowires by directly oxidizing copper substrates has been proposed [14-19].

Using this versatile method, large-scale, vertically aligned CuO nanowires have been successfully produced on various types of copper substrates (sheets, foils, and grids) by directly heating the substrates at 350°C to 700°C in air for several hours. Although numerous studies have been performed on the fabrication of CuO nanowires by the thermal oxidation method, issues related to the growth kinetics and electrical characteristics of CuO nanowires produced under different oxidation conditions have not been extensively explored. Since the growth kinetic data can provide crucial information leading to an understanding of the formation process of CuO nanowires, it is of both fundamental and scientific interests to investigate them further under different experimental conditions.

In the present study, we show the successful fabrication of length-tunable, vertically aligned CuO nanowires on Cu film-coated Si substrates by thermal oxidation in air. The results of a systematic investigation of the growth kinetics, surface morphologies, crystal structures, chemical compositions, and electrical properties of CuO nanowires produced at different oxidation temperatures and times are reported.

## Experimental procedures

Square pieces (8 × 8 mm^2^) were cut from single-crystalline, 10- to 20-Ω cm, p-type (001)Si wafers for use as the deposition substrates in this study. All of these Si substrates were cleaned chemically following a standard procedure and then dipped in a dilute HF solution (HF/H_2_O = 1:20), before being loaded into an electron gun evaporation chamber. A 30-nm-thick Cu thin film with a 30-nm-thick Ti adhesion layer was deposited on the Si substrates to act as the electrode during the subsequent Cu electrodeposition process. The base pressure in the evaporation chamber was better than 1 × 10^-5 ^Pa. The Cu electroplating electrolyte was composed of 0.3 M copper sulfate (CuSO_4_·5H_2_O) and 2.7 M sulfuric acid (H_2_SO_4_). The electrodeposition of pure Cu films on the Cu/Ti film-coated Si substrates was carried out at room temperature using a DC power supply with an applied voltage of 0.2 V. After the Cu electroplating process, the obtained samples were cleaned in a 1 M hydrochloric acid (HCl) solution to remove any surface contaminants and the native oxide layer, then washed with deionized water and blown dry with N_2 _gas. Subsequently, the cleaned Cu-plated Si substrates were oxidized isothermally in an air oven at 400°C to 500°C for 30 to 420 min to grow CuO nanowires. To control the growth process, the air oven temperature was monitored continuously by placing a thermocouple in the vicinity of the Cu film sample during annealing. After the Cu film samples were oxidized, the samples were slowly cooled down in the air oven to room temperature to avoid thermal shocks and achieve crack-free nanowire samples.

The lengths and surface morphologies of the nanowire arrays fabricated on (001)Si substrates at various thermal oxidation temperatures and for various lengths of time were examined by scanning electron microscopy [SEM]. X-ray diffraction [XRD] (Cu Kα radiation, *λ *= 0.154 nm), transmission electron microscopy [TEM], high-resolution TEM [HRTEM], and selected-area electron diffraction [SAED] analysis were carried out for phase identification, atomic structure examination, and crystallography determination. Link ISIS energy-dispersive spectrometers [EDS] attached to the scanning electron microscope and the high-resolution transmission electron microscope were utilized to determine the chemical composition of the produced samples. For TEM and EDS investigations, some of the as-grown nanowires were scratched from the Si substrates and transferred onto carbon film-coated molybdenum mesh grids. The sample was prepared for current-voltage [*I*-*V*] measurement by peeling off a piece of black sheet with CuO nanowires from the surface of an oxidized Cu-plated Si sample and then fixing it to a glass substrate using epoxy glue. Ag paste was then placed at the two ends of the prepared black sheet to act as contact electrodes. The electrical *I*-*V *characteristics of the produced nanowire samples were measured at different temperatures using a Keithley 4200 semiconductor parameter analyzer (Hsinchu, Taiwan).

## Results and discussion

**Figure 1:**
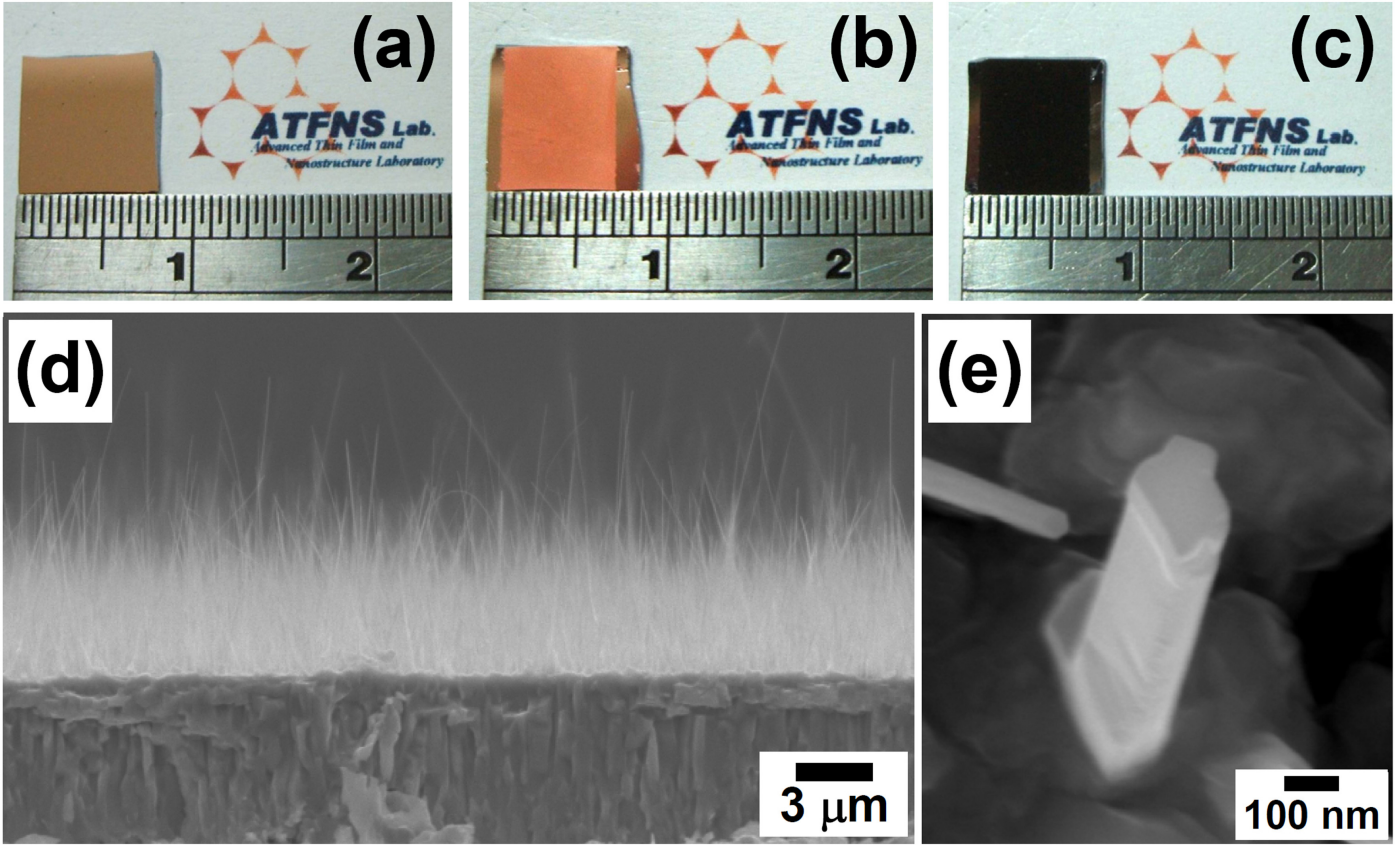
**Photographs of as-deposited Cu/Ti thin film and electrodeposited Cu film and SEM images of nanowires**. Photographs of the (**a**) as-deposited Cu/Ti thin film on a Si substrate and the electrodeposited Cu film-coated Si samples (**b**) before and (**c**) after thermal oxidation annealing. (**d**) A typical cross-sectional SEM image of nanowires grown on an oxidized Cu film-coated Si substrate. (**e**) A typical high-magnification SEM image of an individual nanowire.

**Figure 2:**
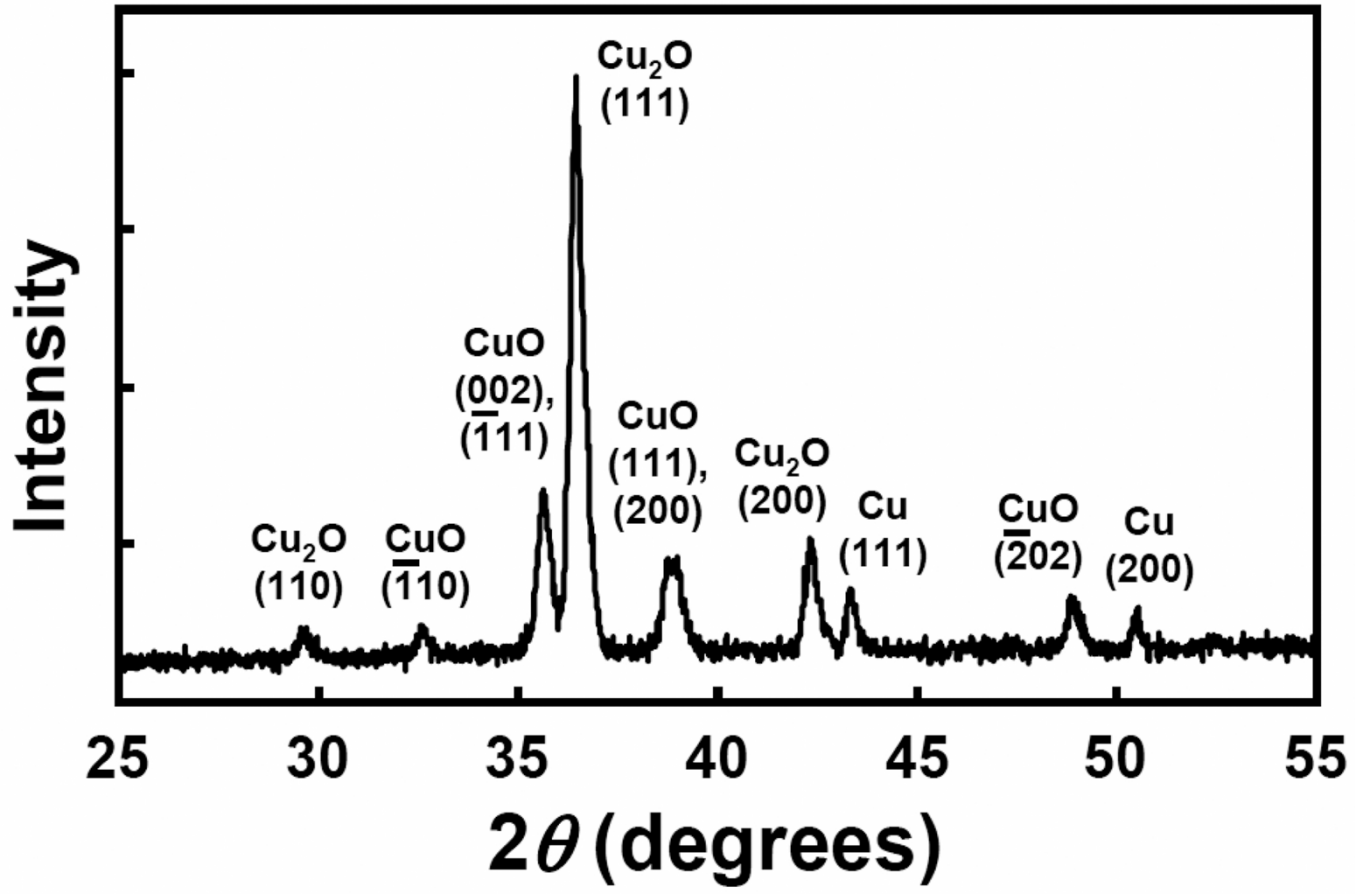
**XRD spectrum**. XRD spectrum of the electrodeposited Cu film-coated Si sample after oxidization in air at 450°C for 180 min.

**Figure 3:**
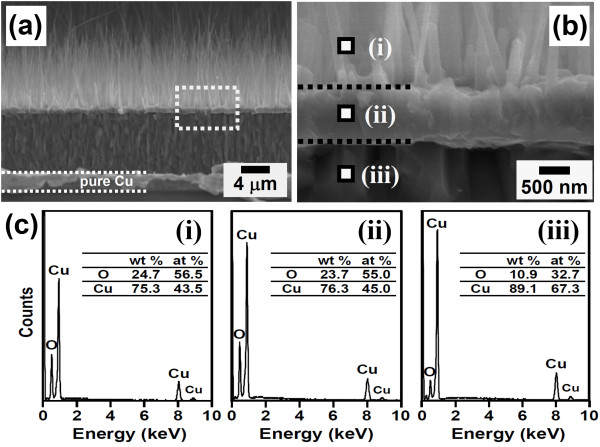
**SEM images and EDS spectra of the oxidized sample**. (**a**) A representative cross-sectional SEM image and (**b**) the corresponding high-magnification image of the thermally oxidized sample; (**c**) EDS spectra taken from the regions marked as (i), (ii) and (iii) shown in (b).

Figure 1a, b, c shows representative photographs of the Cu/Ti film-coated Si sample and the samples after the Cu electroplating process and the thermal oxidation treatment, respectively. The thickness of the electrodeposited pure Cu film was fixed at about 8 μm in this study. It is worthwhile to note that after oxidation annealing, the orange-red color of the electrodeposited Cu film-coated area turned into dark black, see Figure 1b, c. From the SEM observations, it is obvious that high-density, vertically aligned nanowires have grown on the surface of the oxidized (black) area. An example is shown in Figure 1d. The observed black color of the oxidized samples can, therefore, be attributed to light absorption and/or scattering by the nanowire structures. A closer examination, as shown in Figure 1e, reveals that the grown nanowire was heavily faceted, with a relatively flat tip and no catalyst particle could be observed. Figure 2 shows the typical XRD spectrum of the oxidized Cu film-coated Si sample with nanowires. As shown in Figure 2, diffraction peaks corresponding to two copper oxide phases, CuO and Cu_2_O, and a pure Cu phase were detected, indicating that the oxidized sample with nanowires was composed of CuO, Cu_2_O, and pure Cu phases. To clarify the spatial distribution of these phases that formed on the oxidized sample, SEM and EDS analyses were carried out. From cross-sectional SEM observations, a distinct three-layer structure was found to form on the electrodeposited Cu film-coated Si substrate. Representative SEM images are shown in Figure 3a, b. The EDS spectra taken from the regions marked (i), (ii), and (iii) in Figure 3b are shown in Figure 3c. From these EDS analyses, the top nanowire layer and the intermediate thin layer were both identified to be CuO phase, and the bottom thick layer was identified to be Cu_2_O phase. The reaction process and growth mechanism for CuO nanowire formation during the thermal oxidation of Cu films in air have already been extensively investigated and reported in detail in several recent studies [18,20,21]. To further investigate the microstructure and crystallography of the grown nanowires, TEM, SAED, and HRTEM analyses were carried out.

**Figure 4:**
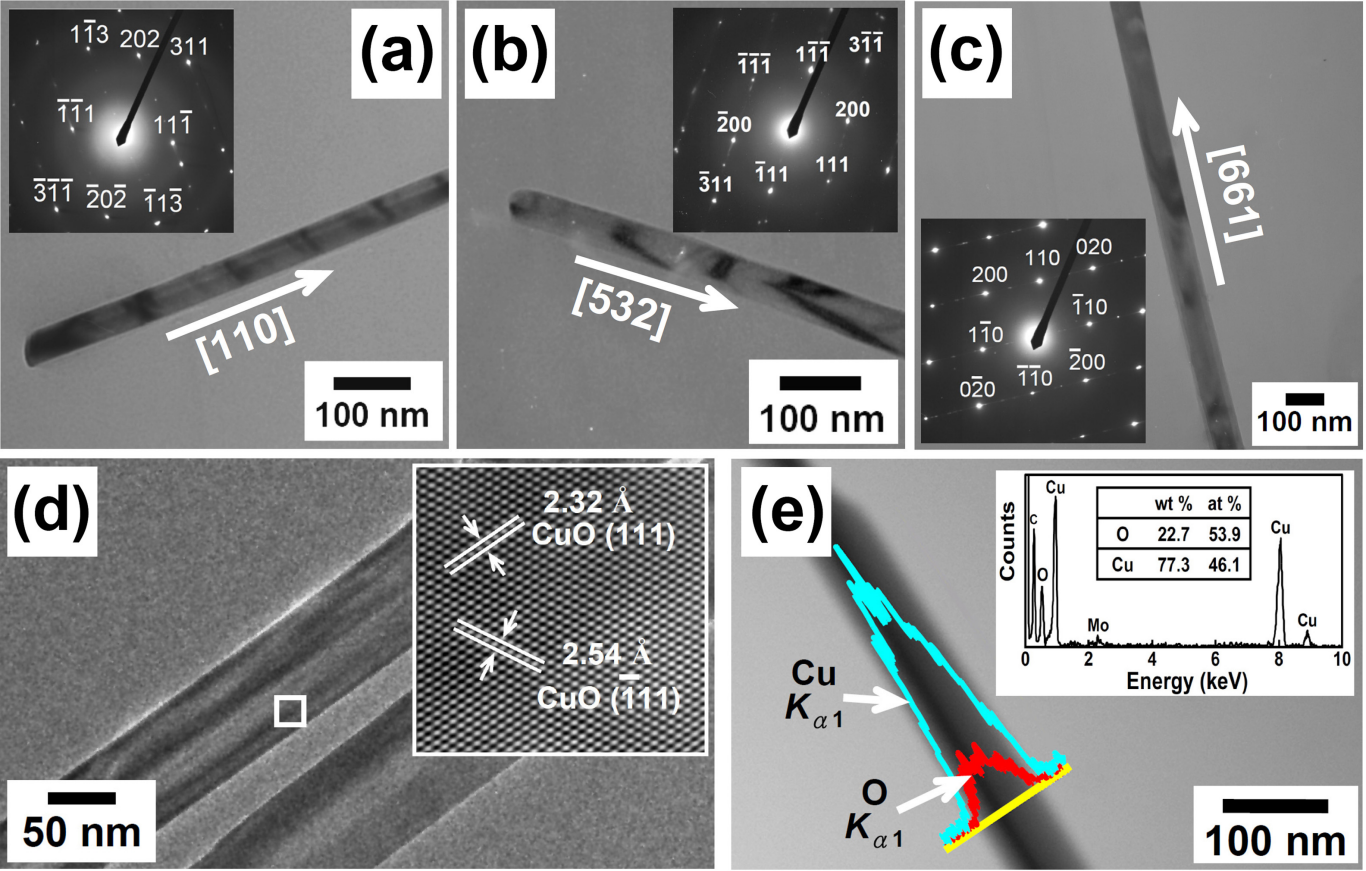
**TEM images, SAED patterns, HRTEM image, and EDS line-scan profile of the CuO nanowires**. (**a**), (**b**), and (**c**) show representative bright-field TEM images of three individual CuO nanowires. The insets show the corresponding indexed SAED patterns. (**d**) TEM image of an individual CuO nanowire. The inset shows the corresponding FFT-filtered lattice images from the outlined region marked in (d). (**e**) TEM image and the EDS line-scan profiles of the CuO nanowire. The inset shows the corresponding spot EDS spectrum.

Figure 4a, b, c shows typical bright-field TEM images of the grown nanowires. The insets show the corresponding indexed SAED patterns. It can be observed from the TEM images that the nanowires have diameters around 30 to 150 nm with smooth and clean surfaces. Analysis of the corresponding SAED patterns clearly indicates that all the produced nanowires were single-crystalline CuO with a monoclinic crystal structure and random axial crystallographic orientations. In addition, pronounced streaking was observed in the SAED patterns, indicating the presence of some planar defects in these CuO nanowires. As can be seen in the HRTEM observation shown in Figure 4d, the measured lattice spacings of 0.232 and 0.254 nm are consistent with the interplanar spacing values for the (111) and ($\bar{1}$11) planes of a pure monoclinic CuO crystal, respectively. Moreover, TEM/EDS analysis further reveals that the nanowires produced were entirely composed of Cu and O, with uniform distributions of Cu and O throughout the nanowires. An example is shown in Figure 4e. The atomic concentration ratio between Cu and O was about 1, which further confirms that the nanowire that formed was indeed a CuO nanowire.

**Figure 5:**
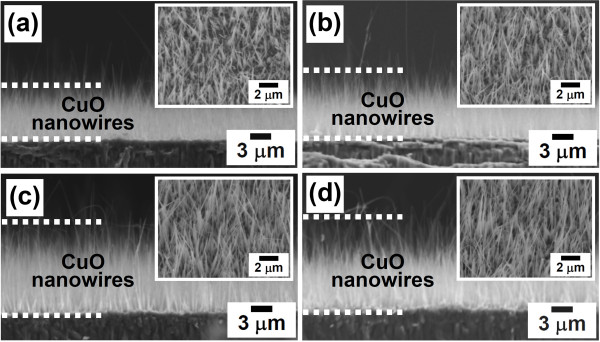
**SEM images of the grown CuO nanowires at different times**. Cross-sectional SEM images of CuO nanowires grown at 475°C for (**a**) 60, (**b**) 120, (**c**) 240, and (**d**) 330 min. The insets show the corresponding top-view SEM images.

**Figure 6:**
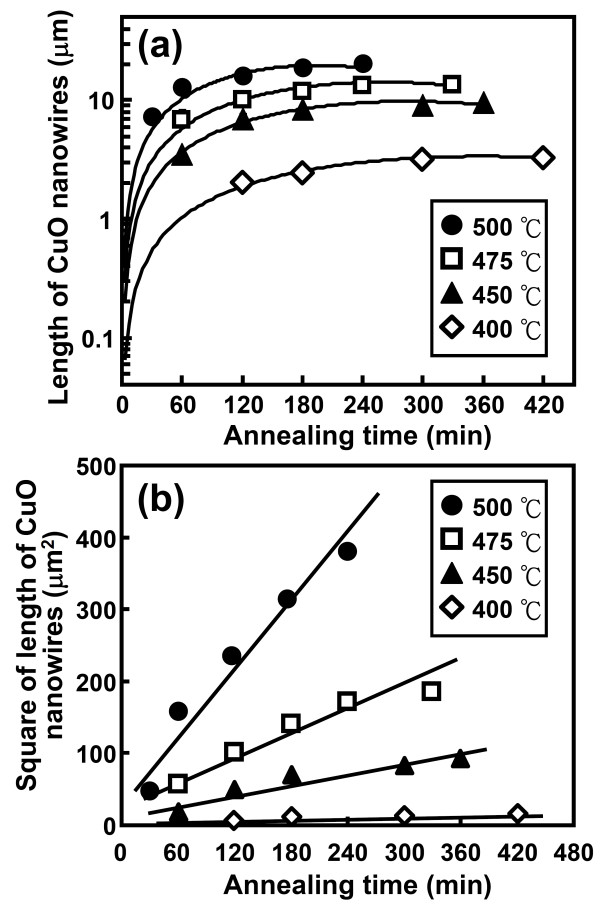
**Average length and square of the length of the CuO nanowires versus annealing time curves**. (**a**) The average length and (**b**) square of the length of CuO nanowires versus annealing time curves for the electrodeposited Cu film-coated Si samples oxidized at 400°C to 500°C.

**Figure 7:**
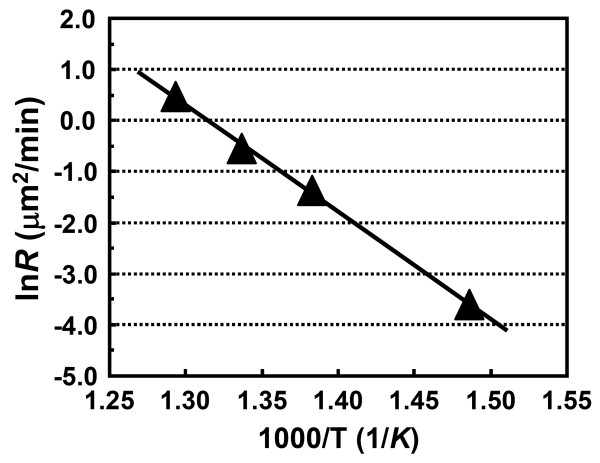
**Arrhenius plot of ln*R *versus the inverse oxidation temperature (1/*T*)**.

Cu film-coated Si samples oxidized in air at 400°C to 500°C for different periods of time were systematically examined by cross-sectional SEM to show the evolution of the length of the CuO nanowires with the oxidation temperature and time. Figure 5a, b, c, d shows the cross-sectional SEM images of CuO nanowires grown at 475°C for 60, 120, 240, and 330 min, respectively. The top right insets in Figure 5 show the corresponding top-view SEM images. From the SEM observations, the CuO nanowires were found to increase in length and density with the duration of the oxidation process. In this study, similar temperature- and time-dependent growth behaviors were also observed for the other Cu film-coated Si samples oxidized at 400°C, 450°C, and 500°C for various periods of time. Figure 6a shows the average lengths of grown CuO nanowires (determined by cross-sectional SEM) as a function of the annealing time for oxidation performed at 400°C to 500°C. In Figure 6a, it is revealed that the lengths of the CuO nanowires that formed at 400°C to 500°C increased parabolically with oxidation time, from 2 to 20 μm. In addition, the growth rate of the CuO nanowires was observed to increase with the oxidation temperature. Figure 6b shows a plot of the square of the CuO nanowire length versus the oxidation time for temperatures from 400°C to 500°C. The relation curves shown in Figure 6b are almost linear, indicating that the growth of the CuO nanowires is diffusion-controlled. Thus, the data for length versus time obtained in Figure 6b can be fitted to the parabolic equation:

(1) $$L^{2}=Rt,$$

where *L *is the length of the CuO nanowire, *t *is the oxidation time, and *R *is the parabolic rate constant. The parabolic rate constant *R *is known to be a function of oxidation temperature *T*, and can be expressed by the Arrhenius equation:

(2) $$R=R_{o}\text{exp}\left(-E_{\text{a}}/kT\right),$$

where *E*_a _is the activation energy, and *R_o _*and *k *are the rate constant and the Boltzmann constant, respectively. The activation energy for the growth of CuO nanowires on Cu film-coated Si substrates can therefore be determined by an Arrhenius plot of the ln(rate constant), ln*R*, versus the reciprocal of oxidation temperature (1/*T*), as shown in Figure 7. The activation energy derived from the slope of the line in Figure 7 is about 174.2 kJ/mole.

**Figure 8:**
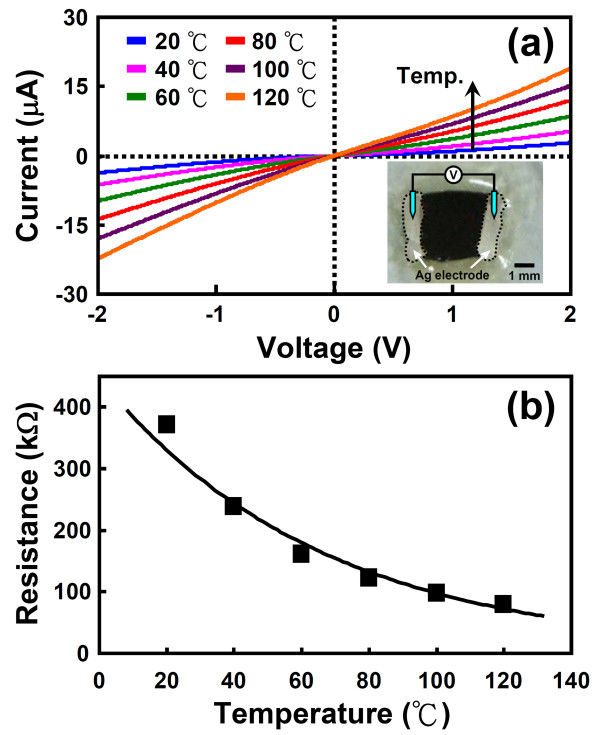
***I*-*V *characteristics and the average electrical resistance of the CuO nanowire sample**. (**a**) *I*-*V *characteristics of the CuO nanowire sample measured at different environmental temperatures. The inset shows a schematic illustration of CuO nanowire device configuration. (**b**) The average electrical resistance of the CuO nanowire sample as a function of environmental temperature.

The electrical behaviors of CuO nanowires were investigated with a standard two-probe technique. The typical *I-V *characteristics of a CuO nanowire sample measured at different temperatures between 20°C and 120°C are shown in Figure 8a. The inset shows a schematic illustration of the CuO nanowire device configuration. Ag paste was placed at the two ends of the CuO nanowire film to act as contact electrodes. The *I*-*V *curves shown in Figure 8a are linear, indicating the establishment of ohmic contact between the CuO nanowire film and the Ag electrodes. The average resistance of the CuO nanowire sample in air at various environmental temperatures could be readily estimated from the slopes of the straight curves presented in Figure 8a. An example is shown in Figure 8b. From Figure 8b, it can be clearly seen that the resistance of the CuO nanowires decreased from 370.4 to 79.4 kΩ as the environmental temperature increased from 20°C to 120°C, which is illustrative of the characteristic behavior of semiconducting materials. This result can be attributed to an increase in the number of carriers at higher temperatures. The temperature-dependent conductive behaviors of the CuO nanowires give rise to potential applications in chemical and gas sensors.

## Summary and conclusions

In summary, the present study demonstrates that by controlling the thermal oxidation temperatures and time, length-tunable, single-crystalline CuO nanowires can be successfully produced on silicon substrates. The growth kinetics, surface morphologies, crystal structures, chemical compositions, and electrical properties of the CuO nanowires produced were investigated.

From the results of TEM, SAED, HRTEM, and EDS analyses, it can be concluded that all the produced nanowires were single-crystalline CuO nanowires with a monoclinic crystal structure, but their axial crystallographic orientations were quite different. Cross-sectional SEM illustrated that for samples oxidized at 400°C to 500°C, the average length of the grown CuO nanowires increased parabolically with the oxidation time. The observed results indicate that the growth of CuO nanowires by thermal oxidation is a diffusion-controlled process. The parabolic growth rate at different oxidation temperatures was also measured. The activation energy for the growth of CuO nanowires was readily derived from an Arrhenius plot to be about 174.2 kJ/mole. On the other hand, the *I*-*V *measurements revealed that the resistance of CuO nanowires decreased significantly from 370.4 to 79.4 kΩ with an increase in the environmental temperature from 20°C to 120°C. The temperature-dependent conductive behavior of the CuO nanowire sample suggests its potential for applications in chemical and gas sensors.

## Competing interests

The authors declare that they have no competing interests.

## Authors' contributions

M-FC carried out the experiments. S-LC supervised the study and drafted the manuscript. All authors read and approved the final manuscript.
